# *N*,*N*′-bis­[(3-hy­droxy-4(4*H*)-oxypyran-2-yl)meth­yl]-*N*,*N*′-dimethyl­ethylene-1,2-diammonium tetra­chloridoplatinate(II) dihydrate

**DOI:** 10.1107/S1600536812040949

**Published:** 2012-10-06

**Authors:** Vieri Fusi, Luca Giorgi, Eleonora Macedi, Paola Paoli, Patrizia Rossi

**Affiliations:** a Department of Basic Sciences and Fundamentals, University of Urbino, I-61029 Urbino, Italy; b Dip. Energetica ‘Sergio Stecco’, University of Firenze, Via S. Marta 3, I-50139 Firenze, Italy

## Abstract

The title compound (C_16_H_22_N_2_O_6_)[PtCl_4_]·2H_2_O, shows anti­proliferative activity in eight tumor cell lines. The asymmetric unit consists of one solvent water mol­ecule on a general position, and one half of each of the polyammonium cation and the tetrachloridoplatinate(II) anion, both of them located on centers of inversion. In the crystal, the cations are connected *via* hydrogen bonding between the carbonyl O atoms and the hydroxyl H atoms into zigzag chains that elongate in the *c-*axis direction. In addition, the carbonyl O atom is hydrogen-bonded to the water mol­ecule which, in turn, inter­acts with the [PtCl_4_]^2−^ anion. Finally, the chains are linked by N—H^+^⋯Cl inter­actions into a three-dimensional network.

## Related literature

For the anti­tumor activity of maltol (systematic name: 3-hy­droxy-2-methyl-4-pyrone) and polyamines, see: Casero & Woster (2001 ▶); Liang *et al.* (2006 ▶); Murakami *et al.* (2006 ▶). For background to the synthesis, solution behaviour, structural properties and biological activity of *N*,*N*′-bis­[(3-hy­droxy-4-pyron-2-yl)meth­yl]-*N*,*N*′-dimethyl­ethylendiamine (Malten), see: Amatori *et al.* (2010 ▶, 2012 ▶).
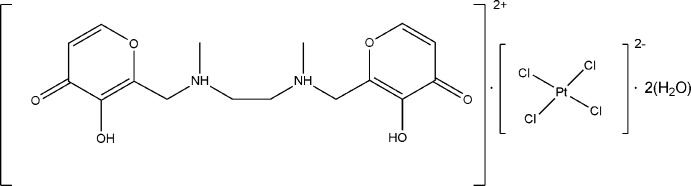


## Experimental

### 

#### Crystal data

(C_16_H_22_N_2_O_6_)[PtCl_4_]·2H_2_O
*M* *_r_* = 711.28
Triclinic, 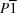

*a* = 6.4775 (4) Å
*b* = 7.0037 (4) Å
*c* = 13.1628 (8) Å
α = 88.810 (5)°
β = 87.033 (5)°
γ = 71.927 (6)°
*V* = 566.92 (6) Å^3^
*Z* = 1
Mo *K*α radiation
μ = 6.71 mm^−1^
*T* = 150 K
0.32 × 0.22 × 0.20 mm

#### Data collection

Oxford Diffraction Xcalibur3 diffractometer
Absorption correction: multi-scan (*CrysAlis PRO*; Oxford Diffraction 2009 ▶) *T*_min_ = 0.164, *T*_max_ = 0.262
9431 measured reflections
2719 independent reflections
2694 reflections with *I* > 2σ(*I*)
*R*_int_ = 0.044

#### Refinement

*R*[*F* ^2^ > 2σ(*F* ^2^)] = 0.023
*wR*(*F* ^2^) = 0.049
*S* = 1.02
2719 reflections
158 parameters
H atoms treated by a mixture of independent and constrained refinement
Δρ_max_ = 1.44 e Å^−3^
Δρ_min_ = −1.14 e Å^−3^

### 

Data collection: *CrysAlis PRO* (Oxford Diffraction 2009 ▶); cell refinement: *CrysAlis PRO*; data reduction: *CrysAlis PRO*; program(s) used to solve structure: *SIR97* (Altomare *et al.*, 1999 ▶); program(s) used to refine structure: *SHELXL97* (Sheldrick, 2008 ▶); molecular graphics: *ORTEP-3* (Farrugia, 1997 ▶); software used to prepare material for publication: *PARST97* (Nardelli, 1995 ▶).

## Supplementary Material

Crystal structure: contains datablock(s) I, global. DOI: 10.1107/S1600536812040949/nc2294sup1.cif

Structure factors: contains datablock(s) I. DOI: 10.1107/S1600536812040949/nc2294Isup2.hkl

Additional supplementary materials:  crystallographic information; 3D view; checkCIF report

## Figures and Tables

**Table 1:** Hydrogen-bond geometry (Å, °)

*D*—H⋯*A*	*D*—H	H⋯*A*	*D*⋯*A*	*D*—H⋯*A*
O1—H1*O*⋯O2^i^	0.73 (4)	1.98 (4)	2.655 (4)	154 (4)
O1*W*—H1*WB*⋯O2^ii^	0.79 (5)	2.07 (5)	2.853 (4)	171 (5)
O1*W*—H1*WA*⋯Cl2	0.84 (5)	2.45 (5)	3.282 (3)	174 (5)
N1—H1*N*⋯Cl1^iii^	0.74 (4)	2.79 (4)	3.380 (3)	139 (4)
N1—H1*N*⋯Cl1^iv^	0.74 (4)	2.79 (4)	3.362 (3)	136 (4)

Symmetry codes: (i) 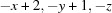; (ii) 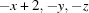; (iii) 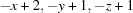; (iv) 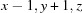.

**Fig. 1.**
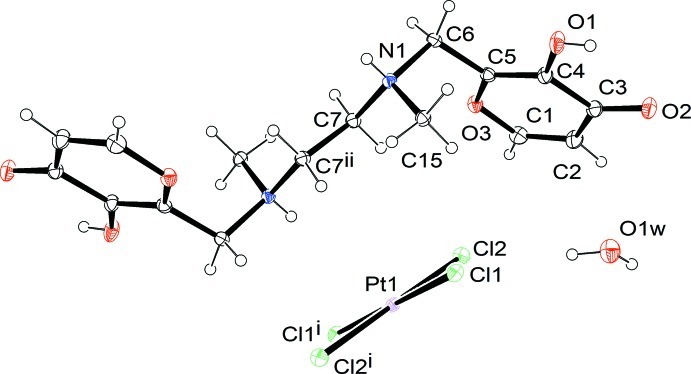
Crystal structure of the title compound with labelling and displacement ellipsoids drawn at the 30% probability level. Symmetry codes: i) = -x + 2, -y, -z + 1; ii) = -x + 1, -y + 1, -z + 1.

**Fig. 2.**
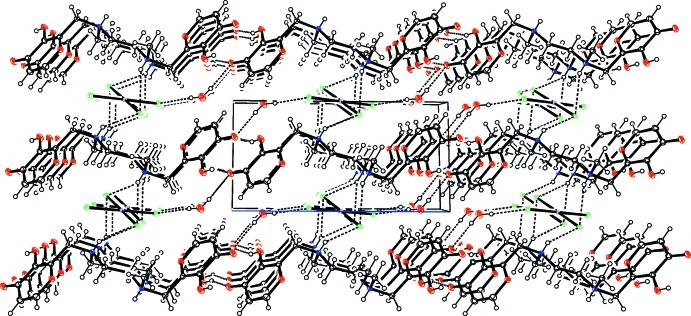
Crystal structure of the title compound with view along the a axis. Intermolecular hydrogen bonding is shown as dashed lines.

## supplementary crystallographic information

### Comment

Maltol (3-hydroxy-2-methyl-4-pyrone) is a natural compound, which exhibits interesting antineoplastic activities (Murakami *et al.*, 2006). At the same time, linear polyamines are also known antitumor agents (Liang *et al.*, 2006; Casero & Woster, 2001). For these reasons we synthesized and studied compound *N*,*N*'-bis((3-hydroxy-4-pyron-2-yl)methyl)-*N*,*N*'-dimethylethylendiamine (Malten) coupling two Maltol units to an aliphatic diamine. Malten has shown antiproliferative activity in eight tumor cell lines (Amatori *et al.*,2010; Amatori *et al.*, 2012). In the asymmetric unit of the title compound half of the polyammonium cation [H_2_Malten]^2+^ and of the tetrachloroplatinate(II) counterion are present, together with a crystallization water molecule. The two halves of each ion are related by a center of symmetry (Fig. 1). The [H_2_Malten]^2+^ polyammonium chain, which joins the two aromatic rings, has an all-*trans* conformation and defines a plane which forms an angle of 65.4 (2)° with each of them. In the crystal lattice the [H_2_Malten]^2+^ cations are each linked by two pairs of complementary O—H···O hydrogen bonds into centrosymmetric dimers, which are further linked into chains along the *c* axis (Fig. 2 and Table 1). Moreover, the carbonyl O atom (O2) is H-bonded to the lattice water molecule, which is also linked to the (PtCl_4_)^2-^ anion by O—H···Cl interactions. Finally, the cations and anions are linked by N—H^+^···Cl interactions.

### Experimental

Malten^.^2HClO_4_ was dissolved in H_2_O, K_2_PtCl_4_ was added and the pH adjusted to 3. Crystals suitable for X-ray analysis formed in one day at room temperature.

### Refinement

The O—H and N—H H atoms were located in the Fourier difference map and refined with varying coordinates isotropic. The C—H H atoms were introduced in calculated position and refined isotropic with *U*_iso_(H) 1.2 times *U*_eq_(C) (1.5 for methyl H atoms).

### Crystal data

**Table:**

(C_16_H_22_N_2_O_6_)[PtCl_4_]·2H_2_O	*Z* = 1
*M**_r_* = 711.28	*F*(000) = 346
Triclinic, *P*1	*D*_x_ = 2.083 Mg m^−^^3^
Hall symbol: -P 1	Mo *K*α radiation, λ = 0.71073 Å
*a* = 6.4775 (4) Å	Cell parameters from 7279 reflections
*b* = 7.0037 (4) Å	θ = 4.1–29.2°
*c* = 13.1628 (8) Å	µ = 6.71 mm^−^^1^
α = 88.810 (5)°	*T* = 150 K
β = 87.033 (5)°	Prismatic, light yellow
γ = 71.927 (6)°	0.32 × 0.22 × 0.20 mm
*V* = 566.92 (6) Å^3^	

### Data collection

**Table:**

Oxford Diffraction Xcalibur3 diffractometer	2719 independent reflections
Radiation source: Enhance (Mo) X-ray Source	2694 reflections with *I* > 2σ(*I*)
Graphite monochromator	*R*_int_ = 0.044
Detector resolution: 16.4547 pixels mm^-1^	θ_max_ = 29.3°, θ_min_ = 4.1°
ω scans	*h* = −8→8
Absorption correction: multi-scan (*CrysAlis PRO*; Oxford Diffraction 2009)	*k* = −9→9
*T*_min_ = 0.164, *T*_max_ = 0.262	*l* = −17→17
9431 measured reflections	

### Refinement

**Table:**

Refinement on *F*^2^	Primary atom site location: structure-invariant direct methods
Least-squares matrix: full	Secondary atom site location: difference Fourier map
*R*[*F*^2^ > 2σ(*F*^2^)] = 0.023	Hydrogen site location: inferred from neighbouring sites
*wR*(*F*^2^) = 0.049	H atoms treated by a mixture of independent and constrained refinement
*S* = 1.02	*w* = 1/[σ^2^(*F*_o_^2^) + (0.0253*P*)^2^] where *P* = (*F*_o_^2^ + 2*F*_c_^2^)/3
2719 reflections	(Δ/σ)_max_ < 0.001
158 parameters	Δρ_max_ = 1.44 e Å^−^^3^
0 restraints	Δρ_min_ = −1.14 e Å^−^^3^

### Special details

**Table:**

Geometry. All s.u.'s (except the s.u. in the dihedral angle between two l.s. planes) are estimated using the full covariance matrix. The cell s.u.'s are taken into account individually in the estimation of s.u.'s in distances, angles and torsion angles; correlations between s.u.'s in cell parameters are only used when they are defined by crystal symmetry. An approximate (isotropic) treatment of cell s.u.'s is used for estimating s.u.'s involving l.s. planes.
Refinement. Refinement of *F*^2^ against ALL reflections. The weighted *R*-factor *wR* and goodness of fit *S* are based on *F*^2^, conventional *R*-factors *R* are based on *F*, with *F* set to zero for negative *F*^2^. The threshold expression of *F*^2^ > 2σ(*F*^2^) is used only for calculating *R*-factors(gt) *etc*. and is not relevant to the choice of reflections for refinement. *R*-factors based on *F*^2^ are statistically about twice as large as those based on *F*, and *R*- factors based on ALL data will be even larger.

### Fractional atomic coordinates and isotropic or equivalent isotropic displacement parameters (Å^2^)

**Table:**

	*x*	*y*	*z*	*U*_iso_*/*U*_eq_	
Pt1	1.0000	0.0000	0.5000	0.01531 (6)	
Cl1	1.25762 (12)	0.12554 (11)	0.42237 (6)	0.01926 (15)	
Cl2	0.82520 (13)	0.02992 (11)	0.34889 (6)	0.02098 (16)	
O1	0.8029 (5)	0.6477 (4)	0.1284 (2)	0.0298 (6)	
O2	0.8598 (4)	0.3386 (4)	−0.00821 (17)	0.0278 (5)	
O3	0.3953 (4)	0.4312 (3)	0.22157 (16)	0.0218 (5)	
N1	0.5757 (4)	0.6487 (4)	0.3876 (2)	0.0169 (5)	
C1	0.4294 (6)	0.2757 (5)	0.1565 (3)	0.0261 (7)	
H1	0.3424	0.1924	0.1649	0.031*	
C2	0.5823 (6)	0.2365 (5)	0.0809 (3)	0.0263 (7)	
H2	0.5998	0.1263	0.0394	0.032*	
C3	0.7197 (5)	0.3601 (5)	0.0625 (2)	0.0224 (7)	
C4	0.6815 (5)	0.5228 (5)	0.1358 (2)	0.0205 (6)	
C5	0.5256 (5)	0.5510 (5)	0.2108 (2)	0.0187 (6)	
C6	0.4731 (5)	0.7161 (5)	0.2869 (2)	0.0190 (6)	
H6A	0.3166	0.7682	0.2984	0.023*	
H6B	0.5233	0.8244	0.2595	0.023*	
C7	0.4568 (5)	0.5266 (5)	0.4470 (2)	0.0181 (6)	
H7A	0.4719	0.4039	0.4103	0.022*	
H7B	0.3033	0.6018	0.4532	0.022*	
C15	0.8152 (5)	0.5442 (5)	0.3735 (2)	0.0181 (6)	
H15A	0.8821	0.6290	0.3351	0.027*	
H15B	0.8389	0.4207	0.3376	0.027*	
H15C	0.8778	0.5159	0.4388	0.027*	
O1W	1.1440 (5)	−0.0251 (4)	0.1423 (2)	0.0343 (6)	
H1N	0.559 (6)	0.742 (6)	0.417 (3)	0.028 (11)*	
H1O	0.888 (7)	0.623 (6)	0.088 (3)	0.024 (11)*	
H1WA	1.062 (9)	−0.020 (8)	0.194 (4)	0.059 (16)*	
H1WB	1.128 (7)	−0.105 (7)	0.104 (3)	0.040 (13)*	

### Atomic displacement parameters (Å^2^)

**Table:**

	*U*^11^	*U*^22^	*U*^33^	*U*^12^	*U*^13^	*U*^23^
Pt1	0.01339 (9)	0.01279 (9)	0.02021 (9)	−0.00476 (6)	0.00072 (6)	−0.00306 (6)
Cl1	0.0169 (4)	0.0185 (4)	0.0241 (4)	−0.0084 (3)	0.0032 (3)	−0.0031 (3)
Cl2	0.0223 (4)	0.0208 (4)	0.0219 (4)	−0.0092 (3)	−0.0040 (3)	−0.0009 (3)
O1	0.0347 (15)	0.0327 (14)	0.0275 (13)	−0.0200 (12)	0.0137 (12)	−0.0108 (11)
O2	0.0310 (14)	0.0309 (13)	0.0237 (12)	−0.0136 (11)	0.0074 (10)	−0.0087 (10)
O3	0.0236 (12)	0.0246 (12)	0.0197 (11)	−0.0112 (10)	−0.0005 (9)	−0.0021 (9)
N1	0.0197 (14)	0.0154 (13)	0.0168 (12)	−0.0074 (11)	0.0002 (10)	−0.0020 (10)
C1	0.0284 (19)	0.0261 (18)	0.0283 (17)	−0.0143 (15)	−0.0056 (15)	0.0001 (14)
C2	0.0310 (19)	0.0242 (17)	0.0271 (17)	−0.0134 (15)	0.0003 (15)	−0.0062 (13)
C3	0.0251 (18)	0.0222 (16)	0.0196 (15)	−0.0064 (13)	−0.0035 (13)	−0.0020 (12)
C4	0.0236 (17)	0.0208 (16)	0.0191 (15)	−0.0096 (13)	−0.0003 (13)	−0.0030 (12)
C5	0.0218 (16)	0.0177 (15)	0.0172 (14)	−0.0065 (13)	−0.0041 (13)	0.0014 (11)
C6	0.0220 (16)	0.0170 (15)	0.0170 (14)	−0.0047 (12)	−0.0027 (12)	0.0018 (11)
C7	0.0171 (15)	0.0176 (15)	0.0200 (15)	−0.0060 (12)	0.0006 (12)	0.0009 (11)
C15	0.0158 (15)	0.0189 (15)	0.0194 (14)	−0.0051 (12)	0.0007 (12)	−0.0008 (11)
O1W	0.0380 (17)	0.0383 (16)	0.0315 (15)	−0.0187 (13)	−0.0001 (13)	−0.0064 (12)

### Geometric parameters (Å, º)

**Table:**

Pt1—Cl1^i^	2.3018 (8)	C2—C3	1.431 (5)
Pt1—Cl1	2.3018 (8)	C2—H2	0.9300
Pt1—Cl2^i^	2.3132 (7)	C3—C4	1.462 (4)
Pt1—Cl2	2.3132 (7)	C4—C5	1.348 (5)
O1—C4	1.345 (4)	C5—C6	1.491 (4)
O1—H1O	0.73 (4)	C6—H6A	0.9700
O2—C3	1.243 (4)	C6—H6B	0.9700
O3—C1	1.356 (4)	C7—C7^ii^	1.526 (6)
O3—C5	1.363 (4)	C7—H7A	0.9700
N1—C15	1.499 (4)	C7—H7B	0.9700
N1—C7	1.501 (4)	C15—H15A	0.9600
N1—C6	1.514 (4)	C15—H15B	0.9600
N1—H1N	0.74 (4)	C15—H15C	0.9600
C1—C2	1.337 (5)	O1W—H1WA	0.84 (5)
C1—H1	0.9300	O1W—H1WB	0.79 (5)
			
Cl1^i^—Pt1—Cl1	180.00 (4)	O1—C4—C3	119.7 (3)
Cl1^i^—Pt1—Cl2^i^	90.28 (3)	C5—C4—C3	121.2 (3)
Cl1—Pt1—Cl2^i^	89.72 (3)	C4—C5—O3	121.9 (3)
Cl1^i^—Pt1—Cl2	89.72 (3)	C4—C5—C6	124.4 (3)
Cl1—Pt1—Cl2	90.28 (3)	O3—C5—C6	113.7 (3)
Cl2^i^—Pt1—Cl2	180.0	C5—C6—N1	112.9 (2)
C4—O1—H1O	115 (3)	C5—C6—H6A	109.0
C1—O3—C5	118.6 (3)	N1—C6—H6A	109.0
C15—N1—C7	113.3 (2)	C5—C6—H6B	109.0
C15—N1—C6	111.5 (2)	N1—C6—H6B	109.0
C7—N1—C6	110.8 (2)	H6A—C6—H6B	107.8
C15—N1—H1N	109 (3)	N1—C7—C7^ii^	111.9 (3)
C7—N1—H1N	107 (3)	N1—C7—H7A	109.2
C6—N1—H1N	106 (3)	C7^ii^—C7—H7A	109.2
C2—C1—O3	123.1 (3)	N1—C7—H7B	109.2
C2—C1—H1	118.5	C7^ii^—C7—H7B	109.2
O3—C1—H1	118.5	H7A—C7—H7B	107.9
C1—C2—C3	121.5 (3)	N1—C15—H15A	109.5
C1—C2—H2	119.2	N1—C15—H15B	109.5
C3—C2—H2	119.2	H15A—C15—H15B	109.5
O2—C3—C2	125.6 (3)	N1—C15—H15C	109.5
O2—C3—C4	120.7 (3)	H15A—C15—H15C	109.5
C2—C3—C4	113.7 (3)	H15B—C15—H15C	109.5
O1—C4—C5	119.1 (3)	H1WA—O1W—H1WB	109 (5)

Symmetry codes: (i) −*x*+2, −*y*, −*z*+1; (ii) −*x*+1, −*y*+1, −*z*+1.

### Hydrogen-bond geometry (Å, º)

**Table:**

*D*—H···*A*	*D*—H	H···*A*	*D*···*A*	*D*—H···*A*
O1—H1*O*···O2^iii^	0.73 (4)	1.98 (4)	2.655 (4)	154 (4)
O1*W*—H1*WB*···O2^iv^	0.79 (5)	2.07 (5)	2.853 (4)	171 (4)
O1*W*—H1*WA*···Cl2	0.84 (5)	2.45 (5)	3.282 (3)	174 (5)
N1—H1*N*···Cl1^v^	0.74 (4)	2.79 (4)	3.380 (3)	139 (4)
N1—H1*N*···Cl1^vi^	0.74 (4)	2.79 (4)	3.362 (3)	136 (4)

Symmetry codes: (iii) −*x*+2, −*y*+1, −*z*; (iv) −*x*+2, −*y*, −*z*; (v) −*x*+2, −*y*+1, −*z*+1; (vi) *x*−1, *y*+1, *z*.
